# Grape Seed Proanthocyanidins Protect Pancreatic β Cells Against Ferroptosis via the Nrf2 Pathway in Type 2 Diabetes

**DOI:** 10.1007/s12011-024-04093-9

**Published:** 2024-02-17

**Authors:** Haiyan Li, Haowei Zhang, Tongling Wang, Liyuan Zhang, Hao Wang, Heng Lu, Ruirui Yang, Yusong Ding

**Affiliations:** 1 Key Laboratory of Environmental Exposome, Xinjiang Medical University, No.393 Xinyi Road, Urumqi, 830011 China; 2 Department of Public Health, Shihezi University School of Medicine, Shihezi, 832000 China; 3 School of Exercise Science, Physical and Health Education, University of Victoria, Victoria, BC V8P 5C2 Canada

**Keywords:** Diabetes mellitus, Type 2, Ferroptosis, GSPE, Iron overload, NF-E2-related factor 2

## Abstract

Pancreatic β cell damage is the primary contributor to type 2 diabetes mellitus (T2DM); however, the underlying mechanism remains nebulous. This study explored the role of ferroptosis in pancreatic β cell damage and the protective effects of grape seed proanthocyanidin extract (GSPE). In T2DM model rats, the blood glucose, water intake, urine volume, HbA1c, and homeostasis model assessment-insulin resistance were significantly increased, while the body weight and the insulin level were significantly decreased, indicating the successful establishment of the T2DM model. MIN6 mouse insulinoma β cells were cultured in high glucose and sodium palmitate conditions to obtain a glycolipid damage model, which was administered with GSPE, ferrostatin-1 (Fer-1), or nuclear factor erythroid 2-related factor 2 (Nrf2) small interfering (si) RNA. GSPE and Fer-1 treatment significantly improved pancreatic β-cell dysfunction and protected against cell death. Both treatments increased the superoxide dismutase and glutathione activity, reduced the malondialdehyde and reactive oxygen species levels, and improved iron metabolism. Furthermore, the treatments reversed the expression of ferroptosis markers cysteine/glutamate transporter (XCT) and glutathione peroxidase 4 (GPX4) caused by glycolipid toxicity. GSPE treatments activated the expression of Nrf2 and related proteins. These effects were reversed when co-transfected with si-Nrf2. GSPE inhibits ferroptosis by activating the Nrf2 signaling pathway, thus reducing β-cell damage and dysfunction in T2DM. Therefore, GSPE is a potential treatment strategy against T2DM.

**Graphical Abstract:**

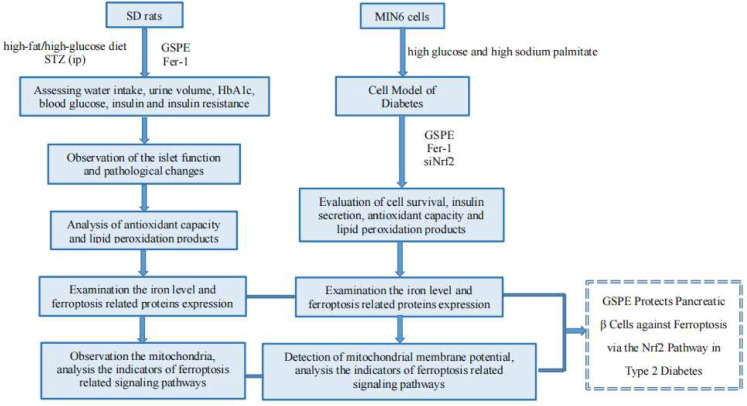

Diabetes mellitus (DM) is a major health concern worldwide owing to its high prevalence and mortality rate. In 2021, approximately 537 million patients (90% type two diabetes mellitus [T2DM]) and 6.7 million DM-related deaths have been reported [1]. Research shows that genetic, environmental, and gut microbiota factors are considered to be the main causes of T2DM, and oxidative stress, apoptosis, endothelial cell damage, inflammation, and autophagy are closely related to the development of T2DM [2]. However, the exact pathogenesis of T2DM remains unknown [3, 4].

Ferroptosis is an iron-dependent cell death accompanied by lipid peroxide accumulation [5]. Previous studies have shown an association between T2DM and ferroptosis, characterized by the iron-dependent production and accumulation of hazardous lipid peroxidation products in cell membranes [6–8]. Sustained high blood sugar not only further damages β cells [9], but also leads to excessive iron accumulation in pancreatic β cells, which in turn induces insulin resistance [10], loss of islet cell mass, and disruption of islet architecture in an iron-dependent manner, eventually causing ferroptosis [11, 12]. Therefore, ferroptosis can be a vital target in treating T2DM.

Nuclear factor erythroid 2-related factor 2 (Nrf2) plays a key role in antioxidation and is a major regulatory factor in inhibiting ferroptosis [13–15]. Nrf2 regulates several genes involved in ferroptosis, such as glutathione peroxidase 4 (GPX4) and cysteine/glutamate transporter (XCT), that inhibit iron overload and lipid peroxidation [15, 16]. The combination of activated Nrf2 protein and antioxidant response element (ARE) initiates the expression of the downstream antioxidant genes (e.g., heme oxygenase 1 (HO-1) and NAD (P) H quinone oxidoreductase 1 (NQO1)), exerting antioxidant activity [17]. Therefore, the activation of the Nrf2 pathway can interrupt ferroptosis.

Grape seed proanthocyanidin (GSPE), one of the most dispersed polyphenols, has free radical scavenging, antioxidative, and anti-inflammatory properties [18–20]. GSPE (grape seed proanthocyanidin extract) has numerous bioactivities and possesses the potential to treat or prevent a wide range of human diseases, and the underlying mechanisms include mediating signaling pathways like NF-κB, MAPK, PI3K/Akt, apoptotic axis, and Nrf-2/HO-1 [21]. GSPE is a natural inhibitor and regulator of iron metabolism and can effectively treat iron overload diseases [14, 22]. A recent study has shown that polyphenols, which act as iron chelators and suppress glutathione (GSH) depletion and lipid peroxidation [22], can protect murine MIN6 pancreatic cells against iron toxicity and erastin-induced ferroptosis [23, 24]. Proanthocyanidins, a family of antioxidants, have been shown to inhibit DM [25]. We have previously demonstrated that GSPE could activate Nrf2 and its downstream antioxidant response genes in the kidney tissues of rats with streptozotocin (STZ)-induced diabetes, improve STZ-induced hyperglycemia, reduce insulin resistance, and slow the progress of diabetic nephropathy [26]. Thus, GSPE might potentially treat ferroptosis as it activates the Nrf2 pathway, the antagonist of ferroptosis. However, the possible effects of GSPE on ferroptosis in T2DM remain undetermined.

Therefore, this study explored the role and mechanism of ferroptosis in T2DM and the potential protective effects of GSPE in antagonizing ferroptosis by activating the Nrf2 pathway. A T2DM rat model was established using STZ, and the glycolipid damage model was set up in mouse insulinoma (MIN6) cells using high glucose and sodium palmitate culture. Our findings might provide novel insights into the role of ferroptosis in the pathogenesis of T2DM and a basis for the utilization of GSPE in treating ferroptosis in patients with T2DM.

## Materials and Methods

### Reagents

GSPE (purity greater than 95%) and ferrostatin-1 (Fer-1) were obtained from Beijing Solarbio Technology (Beijing, China) and MedChemExpress (New Jersey, USA), respectively. Mouse insulinoma (MIN6) cells were acquired from Fuheng Biotechnology Company (Shanghai, China). High-glucose and high-fat (sodium palmitate) kits (KT001) were gathered from the Xi’an Kunchuang Technology (Xi’an, Shannxi, China). SiNrf2 (5′-CCGAAUUACAGUGUCUUAATT-UUAAGACACUGUAA’UUCGGTT-3′) and siRNA negative control (siNc; 5′-UUCUCCGAAGUCACGUTT-ACGUGACACGUUCGGAGAATT-3′) were purchased from GenePharma (Shanghai, China). Anti-4 hydroxynonenal (4-HNE) antibody was acquired from Bioss (MA, USA). Anti-proliferating cell nuclear antigen (PCNA) antibody was obtained from Boster (California, USA). The following antibodies were purchased from Abcam (Cambridge, MA, USA): anti-GPX4, anti-XCT, anti-HO-1, anti-NQO1, and anti-Nrf2. Horseradish peroxidase-conjugated goat anti-mouse/rabbit IgG and mouse anti-β-actin monoclonal antibodies were obtained from ZSGB Biotechnology (Beijing, China). Lipofectamine 2000 was obtained from Invitrogen (Carlsbad, CA, USA). Fitc-labeled goat anti-rabbit IgG (H + L), DAPI, Dual Luciferase Reporter Gene Assay Kit, pRL-TK (Renilla luciferase-thymidine kinase plasmid), and pARE-Luc were acquired from Beyotime Biotechnology (Shanghai, China). The kits for total protein concentration determination (BCA, A045-4), GSH (A006-2), superoxide dismutase (SOD, A001-3), malondialdehyde (MDA, A003-1), and reactive oxide species (ROS; E004-1–1) were obtained from the Nanjing Jiancheng Bioengineering Institute (Nanjing, China). The Cell Counting Kit-8 (CCK8) assay kit (BS350B) and ECL solution (BL520A) were gathered from Biosharp (Anhui, China). The rat transferrin receptor/TFR ELISA Kit (JM-10599R1) was purchased from Jingmei Biotechnology (Jiangsu, China). Lillie Staining Assay kit (G3320) and membrane potential assay kit with JC-1 (M8650) were obtained from Solarbio Technology (Beijing, China). The rat insulin competitive ELISA kit (70-EK3220-24) was purchased from Multi Science (Hangzhou, Zhejiang, China). The rat glycosylated hemoglobin A1c (HbA1c) ELISA Kit (ml-591184) was obtained from the Enzyme-linked Biotechnology Co., Ltd. (Shanghai, China).

### Animals

Healthy Sprague–Dawley (SD) rats (*n* = 110; 8-week-old; weighing 185 ± 25 g), provided by Xinjiang Medical University, were acclimated for 1 week before the investigation. All animal experimental procedures were approved by the Experimental Animal Ethics Committee of the First Affiliated Hospital of Shihezi University School of Medicine (approval no.: A2018-098–01).

### Animal Treatment and Grouping

According to our previously published method, the T2DM model was induced with a high-fat/high-glucose diet and STZ in 80 SD rats [25]. Briefly, rats were fed a high-fat/high-glucose diet for 4 weeks and intraperitoneally injected with STZ in 1% citric acid buffer (40 mg/kg). After 72 h, the fasting blood glucose levels were measured. If the fasting blood glucose levels were > 16.7 mmol/L, the T2DM model was considered successful. The successful rate of model-making was 92.5% (74/80). Another 30 rats were used as controls, which were fed a normal diet and intraperitoneally injected with 1% citric acid buffer solution (pH 4.5).

Rats were divided into control, T2DM, and Fer-1 groups, respectively, with 10 rats in each group to analyze ferroptosis in T2DM. Rats in the control and T2DM groups received intraperitoneal injections of DMSO, while T2DM rats in the Fer-1 group were given intraperitoneal injections of Fer-1 (2.5 μmol/kg) once a day for 2 weeks. Rats were divided into the following groups (*n* = 10 per group): control group, T2DM group, GSPE group (control + 250 mg/kg GSPE), L-GSPE group (T2DM + low dose GSPE [125 mg/kg]), M-GSPE group (T2DM + medium dose GSPE [250 mg/kg GSPE]), and H-GSPE group (T2DM + high dose GSPE [500 mg/kg]) to investigate the effects of GSPE. The rats in the GSPE treatment groups were intragastrically administered GSPE at indicated concentrations, while those in the control and T2DM groups were administered with saline at equal volume. Treatments were administered daily for a total of 12 weeks.

### Sampling

The body weight, fasting blood glucose, water intake, and urine volume of the rats were measured each week and recorded. The rats were anesthetized and then sacrificed at the end of the treatments. Before sacrifice, their blood samples were collected from the rat heart, and the serum was obtained after centrifugation at 3000 × *g*, at 4 °C, for 20 min. Small pieces of the pancreatic tail (1 mm^2^) with abundant pancreatic islets were collected.

### Cell Model of Diabetes

MIN6 cells were cultured in the RPMI 1640 medium containing 10% FBS. The cells were treated with high glucose (20 mM) and high sodium palmitate (300 µM) for 24 h to induce a cell model of diabetes and treated with different doses of GSPE for 24 h and divided into L-GSPE (low dose GSPE, 10 mg/L), M-GSPE (medium dose GSPE, 20 mg/L), and H-GSPE (high dose GSPE, 30 mg/L) groups. Meanwhile, the cell model of diabetes treated with Fer-1 (1 µM) for 24 h served as the positive control group. Cells treated only with high glucose and high sodium palmitate were defined as the GP group.

### Cell Transfection

Cells (1 × 10^5^ cells/mL) were seeded onto the 6-well plates and cultured until 70–80% confluence. siNrf2 or siNC (1.6 µM) was transfected into cells with Lipofectamine 2000. The transfection lasted for 4 or 6 h. Then, the cells were divided into control, control + siNrf2, GP (high glucose [20 mM] and high sodium palmitate [300 µM]), siNrf2 + GP, siNrf2 + GP + GSPE, and siNrf2 + GP + Fer-1 groups. GSPE (20 mg/L) and Fer-1 (1 µM) were treated for 24 h.

### CCK8 Assay

The CCK8 assay was used to assess the cytotoxicity of high glucose, high sodium palmitate, or GSPE on the MIN6 cells. The cells were plated onto the 96-well plates at a density of 7000 cells/well and treated with glucose (20 mM), sodium palmitate (300 µM), or different doses of GSPE (0, 10, 20, and 30 mg/L) for 24 h. Then, 10% CCK8 was added and incubated at 37 °C for 1 h. The cell viability was calculated according to the manufacturer’s instructions.

### Determination of Biochemical Parameters

A conventional glucometer was used to determine the fasting blood glucose levels from the tail vein. The rat glycated HbA1c and rat insulin levels, as well as insulin levels in the cell culture supernatant, were measured using corresponding ELISA kits. The homeostasis model assessment-insulin resistance (HOMA-IR) values were calculated based on fasting glucose and insulin levels according to the following formulation:

$$\mathrm{HOMA}-\mathrm{IR}\;=\;\mathrm{fasting}\;\mathrm{insulin}\times\;\mathrm{fasting}\;\mathrm{blood}\;\mathrm{glucose}/{22.5}$$

### Hematoxylin and Eosin (HE) Staining

HE staining analysis of pancreatic tissues was performed according to our previously published method [23].

### Determination of GSH, MDA, and SOD Levels

The levels of GSH, MDA, and SOD were measured using the corresponding kits according to the manufacturer’s instructions.

### ROS Activity Detection

The MIN6 cells were seeded at a density of 1 × 10^5^ cells per well onto the six-well plates. Treated cells were incubated with the dichloro-dihydro-fluorescein diacetate (DCFH-DA) (1 μM) at 37 °C for 30 min before being rinsed with PBS. A fluorescence microscope was used to observe all fluorescence images of the cells (Leica S6E, Wetzlar, Germany).

### Measurement of Iron Accumulation

The content of transferrin in pancreatic tissue was determined using the rat transferrin ELISA kits, according to the manufacturer’s instructions. According to the kit instructions, the Lillie Staining Assay kit was also used to detect divalent iron deposition in the pancreas and cellular divalent iron. Observations were performed with a fluorescence microscope (Leica S6E, Wetzlar, Germany), while image analysis was conducted using ImageJ software.

### Imaging with the Transmission Electron Microscope

Freshly extracted pancreatic tissues were preserved in 2.5% glutaraldehyde and stored at 4 °C. After washing twice with phosphate buffer (pH 7.4), the tissues were immobilized in 1% osmium tetroxide at room temperature for 1.5 h. Samples were dehydrated and permeated in a graded series of ethanol and acetone solutions, followed by embedment in EPON resin. The samples were polymerized at 37 °C, 45 °C, and 60 °C for 12 h before being sliced into ultra-thin Sects. (70 nm). The sections were stained with uranyl acetate and lead citrate at room temperature for 15 min before imaging on a transmission electron microscope (JEM-1230, JOEL Ltd., Tokyo, Japan). At least five images were taken for each section. Mitochondrial scoring and grading were performed as previously described [27].

### Western Blot Analysis

Western Blot analysis was performed to detect the expressions of PCNA, XCT, GPX4, HO-1, Nrf2, NQO1, and β-actin, according to the previously published method [23].

### Detection of Mitochondrial Membrane Potential

MIN6 cells were seeded onto the 6-well plates at a density of 1 × 10^5^ cells/well, stained with 20 μM JC-1 at 37 °C in the dark for 45 min, and washed twice with JC-1 buffer. Finally, the cells were observed with a fluorescence microscope (Leica S6E, Wetzlar, Germany).

### Immunohistochemical Staining

The expression of 4-HNE, the indicator of lipid peroxidation in rat pancreatic tissues, was detected with immunohistochemistry. Briefly, the tissue sections were incubated with 3% hydrogen peroxidase to inactivate endogenous peroxidase. After antigen retrieval, the sections were incubated with the primary antibody against 4-HNE at 4 °C overnight and then at 37 °C with the corresponding secondary antibody for 50 min. Diaminobenzidine was used for color development. The degree of staining was observed under the microscope (Leica S6E, Wetzlar, Germany).

### ARE Activity Detection

MIN6 cells were seeded onto the 96-well plate. When the cell confluence reached 80–90%, cells were transfected with pARE-Luc and pARE-Luc using Lipofectamine 2000. After incubation at 37 °C for 6 h, the cells were intervened as described above and collected; the luciferase activity was evaluated using a full-wavelength scanning multi-function reader.

### Immunofluorescence Staining

MIN6 cells were seeded onto the six-well plates and intervened as described above, fixed in 4% paraformaldehyde, and permeabilized with 0.1% Triton X-100 at room temperature for 20 min. After washing, the cells were blocked with 5% BSA for 1 h and then incubated with the primary antibody against Nrf2 (1:500) at 4 °C overnight. After washing again, the FITC-labeled goat anti-rabbit IgG H + L (1:1000) was added and incubated at room temperature for 2 h. For nucleus staining, the cells were incubated with DAPI for 3 min. The images were obtained using a fluorescence microscope (Leica S6E, Wetzlar, Germany).

### Statistical Analysis

Data from three independent experiments were expressed as mean ± standard deviation (SD). The SPSS 20.0 software was used for data analysis. One-way ANOVA followed by the Bonferroni test or* t*-test was used to analyze the data. A 95% confidence interval was utilized for all statistical tests. *P* < 0.05 was considered statistically significant.

## Results

### GSPE Improves Pancreatic Function and T2DM Symptoms in Diabetic Rats

**Fig. 1:**
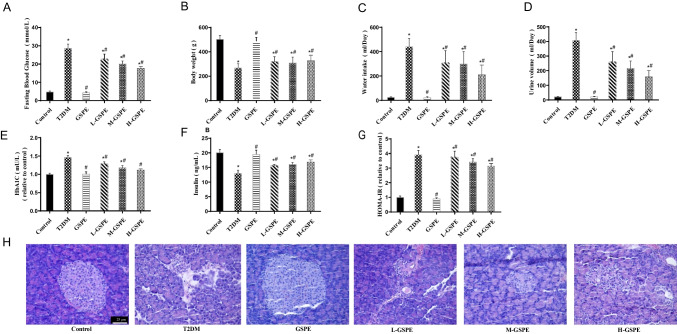
GSPE improves pancreatic function and T2DM symptoms in diabetic rats. Confirmation of the establishment of the diabetes rat model at week 12, as shown by **A** fasting blood glucose levels, **B** body weight, **C** water intake, and **D** urine volume. **E** The hemoglobin A1c (HbA1c) level and **F** insulin level were assessed using ELISA and **G** analysis of homeostasis model assessment-insulin resistance (HOMA-IR) value. HOMA-IR was calculated by fasting insulin × fasting blood glucose/22.5), (H) HE staining of pancreatic islets in diabetic rats (× 400). Data are expressed as mean ± SD (*P < 0.05 vs. control; #*P* < 0.05 vs. T2DM)

To evaluate the establishment of the T2DM model and the effect of GSPE, the rats’ body weight, fasting blood glucose, water intake, and urine volume were monitored. At week 12, the rats in the T2DM group had significantly increased fasting blood glucose (Fig. 1A), water intake (Fig. 1C), and urine volume (Fig. 1D), with significantly decreased body weight (Fig. 1B) compared with the control group (*P* < 0.05). These typical symptoms of T2DM indicated the successful establishment of the T2DM model. Compared with the T2DM group, GSPE administration in L-/M-/H-GSPE groups significantly reduced blood glucose, water intake, and urine volume while elevating body weight (*P* < 0.05). Moreover, compared with the control group, rats in the T2DM group showed significantly increased HbA1c levels (Fig. 1E) and decreased insulin levels (Fig. 1F), and thus significantly increased HOMA-IR value (Fig. 1G) (*P* < 0.05). However, these changes in L-/M-/H-GSPE groups were reversed by GSPE (*P* < 0.05). Additionally, HE staining showed markedly depleted levels of Langerhans islets in the T2DM group compared with the control group (Fig. 1H). However, GSPE administration in the L-/M-/H-GSPE groups provided better protection against islet injury, especially regarding islet size and structure, compared with the T2DM group (Fig. 1H). No significant differences between the control and GSPE groups were observed.

### Ferroptosis Is Activated in Diabetic Rats

**Fig. 2:**
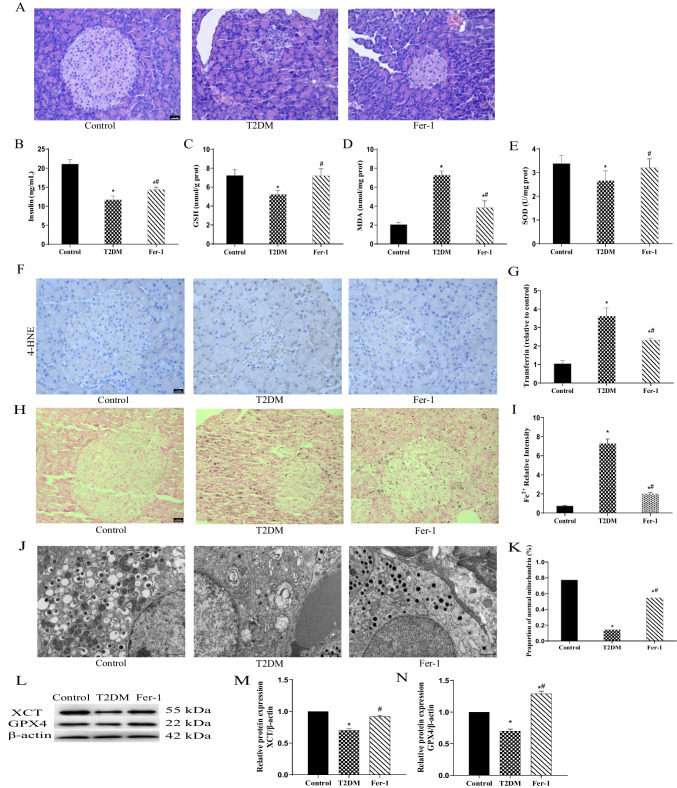
Ferroptosis of pancreatic tissue is activated in T2DM rats. **A** HE staining of rat pancreatic tissues (magnification × 400). **B** Insulin levels were measured using rat-specific ELISA kits. **C**–**E** Glutathione (GSH) (**C**), malondialdehyde (MDA) (**D**), and superoxide dismutase (SOD) levels (**E**) were detected in pancreatic homogenates. **F** The protein expression of 4-HNE detected using immunohistochemical staining (magnification × 400). **G** Quantification of transferrin using rat-specific ELISA kits. **H** Lillie staining was used to detect ferrous iron content (blue) in pancreatic tissue (magnification × 400). **I** quantification of the ferrous iron content (× 400). **J** Transmission electron microscope observation of mitochondrial structures in the pancreas. **K** The proportion of normal mitochondria. **L** XCT and GPX4 protein expression levels in pancreatic tissue detected using western Blot analysis. **M**, **N** Quantified XCT (**M**) and GPX4 (**N**) protein levels. Data are expressed as mean ± SD (*P < 0.05 vs. Control; #*P* < 0.05 vs. T2DM)

We assessed the involvement of ferroptosis in the pancreatic damage of T2DM. HE staining showed that the structure of pancreatic tissue in diabetic rats of the T2DM group was markedly damaged, while in the control group, islet composition was complete (Fig. 2A). Treatment with Fer-1 alleviated the damages induced by T2DM. Furthermore, the Fer-1 group had significantly increased insulin levels compared with the T2DM group (Fig. 2B; *P* < 0.05). In diabetic rats of the T2DM group, the GSH (Fig. 2C) and SOD (Fig. 2E) levels were reduced remarkably. In contrast, the MDA levels (Fig. 2D) were significantly increased compared with the control group (*P* < 0.05). Importantly, Fer-1 rescued the GSH and SOD levels and reduced the MDA accumulation (Fig. 2C–E) (*P* < 0.05). Immunohistochemistry showed that the protein level of 4-HNE was elevated in diabetic rats, while the Fer-1 therapy reduced its level (Fig. 2F). In the T2DM group, transferrin was elevated in pancreatic tissues (Fig. 2G). Lillie staining showed iron deposition at the center of the islets of the T2DM group, indicating iron overload (Fig. 2H, I). Notably, Fer-1 could reverse these effects (Fig. 2G–I). As shown in the mitochondrial ultrastructural micrographs (Fig. 2J), mitochondrial membrane rupture and cristae loss were observed in the islet cells of diabetic rats of the T2DM group, with a reduced proportion of normal mitochondria (Fig. 2K). Fer-1 mitigated the changes in mitochondria. Western blot found that the protein expression levels of GPX4 and XCT were decreased in the T2DM group (Fig. 2L–N). These effects were abolished following Fer-1 treatment (Fig. 2L–N).

### GSPE Improves Pancreatic Ferroptosis in Diabetic Rats

**Fig. 3:**
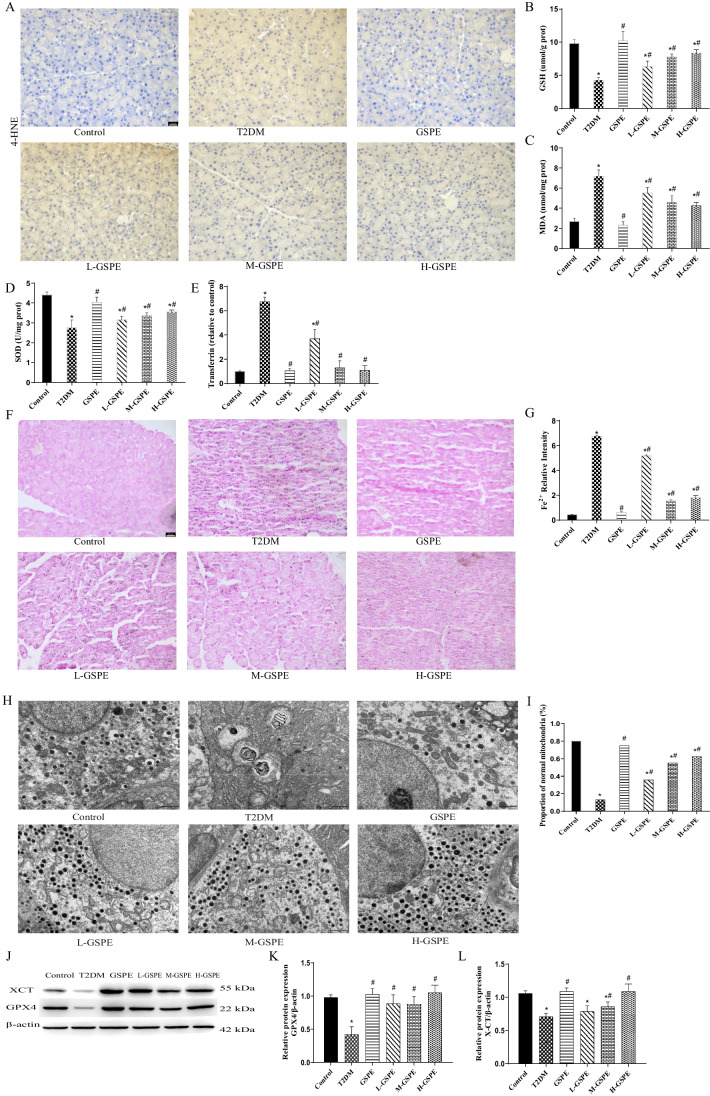
GSPE improves pancreatic ferroptosis in diabetic rats. **A** Immunohistochemical staining of 4-HNE (magnification × 400). **B**–**D** The levels of glutathione (GSH) (**B**), malondialdehyde (MDA) (**C**), and superoxide dismutase (SOD) (**D**) in pancreatic homogenates were assessed. **E** Transferrin was measured using rat-specific ELISA kits. **F** Lillie staining was used to detect ferrous iron content (blue) in pancreatic tissue (magnification × 400). **G** Quantified ferrous iron content. **H** The mitochondrial structure was observed with a transmission electron microscope. **I** The proportion of normal mitochondria. **J** Representative western blot results of GPX4 and XCT proteins. **K**, **L** Quantified levels of GPX4 and XCT proteins. Data are expressed as mean ± SD (*P < 0.05 vs. Control; #*P* < 0.05 vs. T2DM)

Next, we evaluated the effect of GSPE on ferroptosis in T2DM. Compared with the control group, the T2DM group showed increased levels of 4-HNE (Fig. 3A) and MDA (Fig. 3C) and significantly reduced levels of GSH (Fig. 3B) and SOD (Fig. 3D). However, these changes were reversed in the L-/M-/H-GSPE groups (Fig. 3A–D; *P* < 0.05). The transferrin and divalent iron levels in pancreatic tissue were determined to verify whether GSPE can improve iron overload. The results showed that the levels of transferrin in the pancreatic tissue were significantly increased in the T2DM group (Fig. 3E). The iron deposition indicated iron overload (Fig. 3F, G). These effects were reversed following GSPE treatment (Fig. 3E–G; *P* < 0.05). Transmission electron microscopy showed that GSPE improved the mitochondrial cristae loss and outer membrane rupture in the mitochondria (Fig. 3H), with a significantly increased normal mitochondria count (Fig. 3I). In addition, the protein levels of GPX4 and XCT in the L-/M-/H-GSPE groups were significantly increased compared with the T2DM group (Fig. 3J–L; *P* < 0.05).

### Protective Effect of GSPE on Glucolipotoxicity-Induced Ferroptosis in MIN6 Cells

**Fig. 4:**
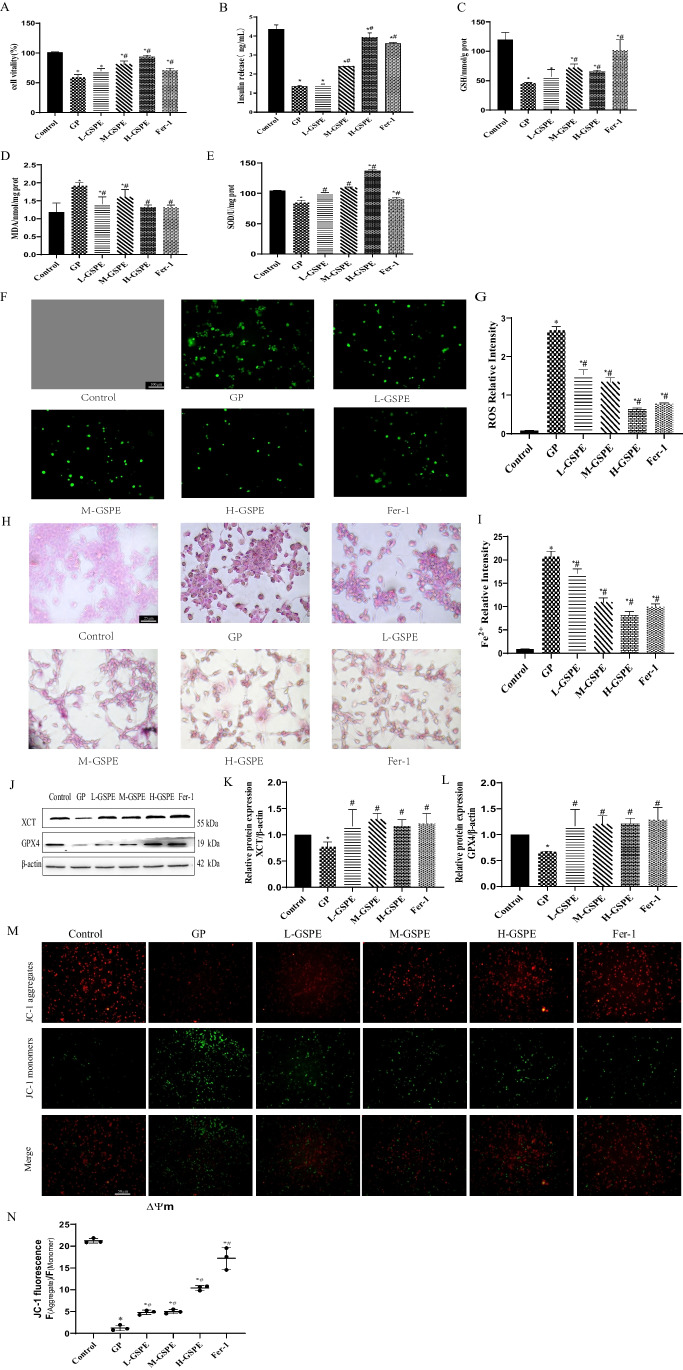
GSPE alleviates ferroptosis-related injury induced by high glucose and high sodium palmitate in MIN6 cells. **A** Cell viability in the indicated groups. **B** Effects of GSPE on glucose-stimulated insulin secretion. **C**–**E** GSH, SOD, and MDA levels determined in the MIN6 cells. **F** ROS levels detected using (DCFH-DA) staining (magnification × 100). **G** Relative ROS intensity. **H** Lillie staining was used to detect ferrous iron content (blue) in MIN6 cells (magnification × 400). **I** Quantified ferrous iron content. **J**–**L** XCT and GPX4 protein expression levels detected using western blot. Representative and quantitative results are shown. **M** Relative JC-1 fluorescence. **N** JC-1 staining of the mitochondrial membrane potential (× 200). Data are expressed as mean ± SD (*P < 0.05 vs. control; #*P* < 0.05 vs. GP)

In vitro, GSPE improved cell viability and could protect cells from glycolipid toxicity (Fig. 4A). Furthermore, as shown in Fig. 4B, the insulin level of the MIN6 cells was significantly decreased in the GP group, and the intervention of GSPE and Fer-1 improved the insulin secretion of the MIN6 cells. Furthermore, the levels of MDA, GSH, and SOD were determined (Fig. 4C–E). Similar to the results in diabetic rats, GSPE and Fer-1 treatment up-regulated GSH and SOD but down-regulated MDA in MIN6 cells with glycolipid injury. In addition, DCFH-DA staining showed that the L-/M-/H-GSPE and Fer-1 groups had significantly lower ROS levels than the GP group (Fig. 4F, G). As shown in Fig. 4H, I, GSPE and Fer-1 alleviated cellular iron accumulation. Western blot found that compared with the GP group, GSPE and Fer-1 increased the protein levels of GPX4 and XCT (Fig. 4J–L). JC-1 staining revealed that the red fluorescence intensity of the cells in the GP group was significantly reduced, while the green fluorescence intensity was significantly increased, suggesting a reduction in mitochondrial membrane potential, leading to increased cell death. However, the Fer-1 and GSPE treatment reversed this effect (Fig. 4M, N).

### GSPE Protects Against Glucolipotoxicity-induced Ferroptosis Through Activating the Nrf2 Pathway

**Fig. 5:**
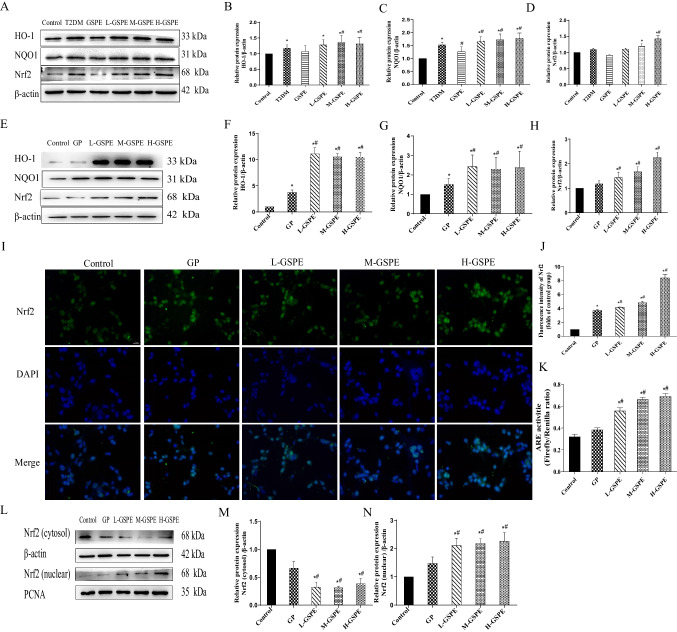
GSPE protects against glucolipotoxicity-induced ferroptosis by activating the Nrf2 pathway. **A**–**H** HO-1, NQO1, and Nrf2 expression levels from MIN6 cells determined using western blot and quantified. **I**, **J** Immunofluorescence analysis of Nrf2. **K** Analysis of ARE activity. **L**–**N** Cytoplasmic and nuclear Nrf2 proteins detected and quantified using western blot. Data are expressed as mean ± SD (*P < 0.05 vs. control; #*P* < 0.05 vs. GP)

Nrf2 plays an essential role in ferroptosis regulation. To determine whether the protective effect of GSPE on ferroptosis induced by glycolipid toxicity is achieved by activation of the Nrf2 pathway, the expression of Nrf2, NQO1, and HO-1 in MIN6 cells and rat pancreatic tissues was evaluated. As shown in Fig. 5A–H, different doses of GSPE treatment elevated the protein levels of Nrf2, NQO1, and HO-1 in MIN6 cells with glycolipid injury. Immunofluorescence staining showed that GSPE significantly increased the Nrf2 intensity in the cell nucleus, suggesting that GSPE promotes the nuclear translocation of Nrf2 (Fig. 5I–J). This result was confirmed by western blot analysis of Nrf2 in the cytoplasm and the nucleus (Fig. 5L–N; *P* < 0.05). The results of the luciferase analysis revealed that GSPE significantly increased the ARE activity (Fig. 5K; *P* < 0.05). Therefore, GSPE activated the Nrf2 signaling pathway in vitro and in vivo.

### siNrf2 Exacerbates Ferroptosis Induced by Glucolipotoxicity in MIN6 Cells

**Fig. 6:**
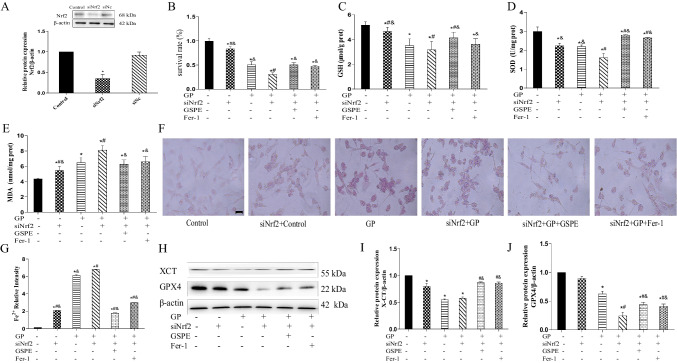
SiNrf2 exacerbates ferroptosis induced by glucolipotoxicity in MIN6 cells. **A** Nrf2 protein expression detected and quantified using western blot. **B** Cell viability measured in MIN6 cells after silencing Nrf2 using the CCK8 assay. **C**–**E** GSH, SOD, and MDA levels determined in MIN6 cells after silencing Nrf2 genes. **F**, **G** Lillie staining was used to detect and quantify ferrous iron content (blue) in MIN6 cells after silencing Nrf2 genes (× 400). **H**–**J** XCT and GPX4 protein expression levels detected and quantified using western blot analysis. Data are expressed as mean ± SD (*P < 0.05 vs. Control; #*P* < 0.05 vs. GP; &*P* < 0.05 vs. siNrf2 + GP)

siNrf2 transfection was performed to knock down Nrf2 expression. As shown in Fig. 5A, Nrf2 expression significantly decreased after the transfection of siNrf2. GSPE increased the cell viability when co-transfected with siNrf2 in MIN6 cells (Fig. 5B). Meanwhile, the GSH, SOD, and MDA contents were estimated in the glucolipotoxicity-induced MIN6 cells after siNrf2 transfection. Compared with the siNrf2 group, the GSH and SOD contents of the GP group were reduced, while the MDA level was increased. GSPE reversed the changes in these indicators (Fig. 5C–E). As shown in Fig. 5F, G, the Fe^2+^ content was increased in the GP group, while GSPE treatment alleviated cellular iron accumulation. Western blot showed that compared with the GP group, GSPE treatment increased the protein levels of GPX4 and XCT (Fig. 5H–J and 6).

## Discussion

Ferroptosis has attracted significant interest since its first discovery in 2012, yet, up to date, only a few studies have explored the relationship between T2DM and ferroptosis. This study investigated the role of ferroptosis in STZ-induced pancreatic injury in T2DM rats and the effect of GSPE on ferroptosis in pancreatic β cells. Our results indicated that ferroptosis involved the β-cell death in glucolipotoxicity-induced pancreatic injury and that GSPE improved the STZ-induced pancreatic oxidative damages and functional and pathological changes in T2DM rats. In addition, GSPE reversed the changes in the expression of GPX4 and XCT in ferroptosis and increased the expression of Nrf2 and its downstream proteins. These results suggest that GSPE may activate the Nrf2 signaling pathway, which antagonizes glucolipotoxicity-induced oxidative damage and inhibits ferroptosis in β cells, making it a possible treatment for ferroptosis in T2DM.

Research has shown that the mechanism of the health effects of the essential trace elements or potentially toxic trace elements involves ferroptosis, which depends on the intracellular iron level, accumulation of lipid ROS, and loss of activity of the lipid-repairing enzyme GPX4 [28]. In recent years, increasing evidence has shown that iron overload is a critical initiator of ferroptosis and a significant risk factor for T2DM [29, 30]. Our results are consistent with the literature that the glycolipid toxicity up-regulated the expression of transferrin and ferritin, thereby increasing the pancreatic iron deposition in T2DM rats and MIN6 cells.

Additionally, studies have shown that diabetes induces ROS overproduction and interferes with the antioxidant defense system by modifying the activity of antioxidant enzymes [23, 31]. Excessive ROS can attack unsaturated fatty acids in the biofilm, leading to lipid peroxidation and, ultimately, lipid peroxide formation. Moreover, MDA expression reflects the degree of lipid peroxidation and oxidative damage to cells [32]. Our results showed that diabetic rats and MIN6 cells with glycolipid injury showed significantly decreased GSH contents and SOD activity and increased ROS and MDA levels, indicating that ferroptosis was involved in pancreatic islet β-cell injury caused by glycolipid toxicity via increased intracellular iron concentration and accumulated lipid peroxides.

Furthermore, GPX4 is the primary enzyme preventing ferroptosis. GPX4 knockout can induce cell death in a pathologically relevant form of ferroptosis [33], while GPX4 overexpression can delay motor neuron disease in SOD1G93A mice by inhibiting ferroptosis [34]. Evidence suggests that insulin-producing β cells are susceptible to ferroptosis unless protected by GPX4 [35]. In addition, the levels of both XCT and GPX4 proteins were down-regulated in T2DM rat pancreas and MIN6 cells with glycolipid injury. Transmission electron microscopy revealed condensed mitochondrial membrane densities (smaller than normal mitochondria) and diminished mitochondrial cristae and outer membrane rupture, in line with the findings of Li et al. [23]. Fer-1 treatment significantly improved the above indices in diabetic rats and MIN6 cells with glycolipid injury in vivo and in vitro, further suggesting the presence of ferroptosis in β cells and T2DM. Therefore, targeted inhibition of ferroptosis may be a promising treatment for T2DM.

Considering the antioxidant effects and iron chelation properties of GSPE [14, 36], we speculated that GSPE could alleviate ferroptosis in β cells and oxidative damage in the pancreas caused by glycolipid toxicity. As expected, GSPE treatment improved the blood glucose level, iron content, accumulation of lipid peroxides, and islet injury in the diabetic model; the same effect was observed in the MIN6 cells with glycolipid injury. In addition, GSPE reversed the above indicators related to ferroptosis in vivo and in vitro. Zhou et al. have reported similar findings, which showed that proanthocyanidins could improve motor function in mice with spinal cord injury by inhibiting ferroptosis [14]. Therefore, our findings suggest that the protective effect of GSPE on β cell damage caused by glucolipotoxicity is mediated, at least in part, by ferroptosis inhibition.

Moreover, GSPE might regulate the Nrf2 signaling pathway to inhibit ferroptosis. Nrf2 is a stress-inducible transcription factor. Several proteins and enzymes are responsible for preventing lipid peroxidation and, thus, inhibiting ferroptosis are Nrf2 target genes [37]. Nrf2 has been shown to regulate dozens of genes involved in ferroptosis regulation, such as GPX4 and XCT [21, 38]. The activated Nrf2 protein combines with the antioxidant reaction element (ARE), thus initiating the expression of the downstream antioxidant genes, HO-1 and NQO1, resulting in an antioxidant effect [39–41]. Our previous study has shown that GSPE activated the expression of HO-1, NQO1, and Nrf2 in the kidney tissue of T2DM rats [42]. The results of this study indicated that GSPE treatment significantly increased nuclear Nrf2, HO-1, and NQO1 protein levels and GSH and SOD levels while reducing ROS and MDA levels. These results revealed that GSPE might protect the pancreas from oxidative stress by activating the Nrf2 signaling pathway to inhibit ferroptosis in T2DM. To further verify the role of Nrf2, we performed Nrf2 knockdown by siRNA. Notably, siNfr2 remarkably increased glucolipotoxicity-induced cytotoxicity, oxidative stress, iron overload, and ferroptosis in MIN6 cells with glycolipid injury. Meanwhile, these damages were reversed by GSPE, suggesting that GSPE exerts a protective effect against β cells from ferroptosis by activating Nrf2.

However, this study has some limitations. Due to time and funding constraints, we only selected MIN6 cells for the experiment instead of rat islet–isolated or human islet cells. An extended investigation using rat islet–isolated or human islet cells may be valuable to support the current findings. In addition, when verifying the Nrf2 signaling pathway, we did not use Nrf2 gene knockout rats; therefore, further investigations are warranted. Nevertheless, this study demonstrated that GSPE could effectively protect β cells against T2DM-induced ferroptosis via the activation of the Nrf2 pathway. Therefore, GSPE can potentially be used to treat T2DM-induced ferroptosis, which has significant clinical implications.

## Conclusion

This study investigated how ferroptosis affects STZ-induced pancreatic injury in T2DM rats and whether GSPE could inhibit ferroptosis in pancreatic β cells. Our results showed that (i) high glucose and sodium palmitate lead to ferroptosis in pancreatic β cells of T2DM rats and MIN6 cells; (ii) GSPE increased the expression of GPX4 and XCT, effective ferroptosis inhibitors that can improve T2DM; and (iii) GSPE significantly reduced ferroptosis and improved T2DM and β-cell viability through the Nrf2 pathway, in vivo and in vitro. This study provides evidence of the protective role of GSPE in T2DM by inhibiting ferroptosis. Our findings demonstrate the important role of ferroptosis in the pathogenesis of T2DM both in vivo and in vitro and provide a basis for using GSPE in clinical settings.

## Data Availability

The datasets generated and/or analyzed in this study are available from the corresponding author at a reasonable request.
